# The Potential Role of the cABR in Assessment and Management of Hearing Impairment

**DOI:** 10.1155/2013/604729

**Published:** 2013-01-30

**Authors:** Samira Anderson, Nina Kraus

**Affiliations:** ^1^Auditory Neuroscience Laboratory, Northwestern University, Evanston, IL 60208, USA; ^2^Department of Communication Sciences, Northwestern University, Evanston, IL 60208, USA; ^3^Department of Hearing and Speech Sciences, University of Maryland, 0100 Lefrak Hall, College Park, MD 20742, USA; ^4^Department of Neurobiology and Physiology, Northwestern University, Evanston, IL 60208, USA; ^5^Department of Otolaryngology, Northwestern University, Evanston, IL 60208, USA

## Abstract

Hearing aid technology has improved dramatically in the last decade, especially in the ability to adaptively respond to dynamic aspects of background noise. Despite these advancements, however, hearing aid users continue to report difficulty hearing in background noise and having trouble adjusting to amplified sound quality. These difficulties may arise in part from current approaches to hearing aid fittings, which largely focus on increased audibility and management of environmental noise. These approaches do not take into account the fact that sound is processed all along the auditory system from the cochlea to the auditory cortex. Older adults represent the largest group of hearing aid wearers; yet older adults are known to have deficits in temporal resolution in the central auditory system. Here we review evidence that supports the use of the auditory brainstem response to complex sounds (cABR) in the assessment of hearing-in-noise difficulties and auditory training efficacy in older adults.

## 1. Introduction

In recent years, scientists and clinicians have become increasingly aware of the role of cognition in successful management of hearing loss, particularly in older adults. While it is often said that “we hear with our brain, not just with our ears,” the focus of the typical hearing aid fitting continues to be one of providing audibility. Despite evidence of age-related deficits in temporal processing [1–6], abilities beyond the cochlea are seldom measured. Moreover, when auditory processing is assessed, behavioral measures may be affected by reduced cognitive abilities in the domains of attention and memory [7, 8]; for example, an individual with poor memory will struggle to repeat back long sentences in noise. The assessment and management of hearing loss in older adults would be enhanced by an objective measure of speech processing. The auditory brainstem response (ABR) provides such an objective measure of auditory function; its uses have included evaluation of hearing thresholds in infants, children, and individuals who are difficult to test, assessment of auditory neuropathy, and screening for retrocochlear function [9]. Traditionally, the ABR has used short, simple stimuli, such as pure tones and tone bursts, but the ABR has also been recorded to complex tones, speech, and music for more than three decades, with the ABR's frequency following response (FFR) reflecting the temporal discharge of auditory neurons in the upper midbrain [10, 11]. Here, we review the role of the ABR to complex sounds (cABR) in assessment and documentation of treatment outcomes, and we suggest a potential role of the cABR in hearing aid fitting.

## 2. The cABR Approach

The cABR provides an objective measure of subcortical speech processing [12, 13]. It arises largely from the inferior colliculus of the upper midbrain [14], functioning as part of a circuit that interacts with cognitive, top-down influences. Unlike the click-evoked response, which bears no resemblance to the click waveform, the cABR waveform is remarkably similar to its complex stimulus waveform, whether a speech syllable or a musical chord, allowing for fine-grained evaluations of timing, pitch, and timbre representation. The click is short, nearly instantaneous, or approximately 0.1 ms, but the cABR may be elicited by complex stimuli that can persist for several seconds. The cABR's response waveform can be analyzed to determine how robustly it represents different segments of the speech stimulus. For example, in response to the syllable /da/, the onset of the cABR occurs at approximately 9 ms after stimulus onset, which would be expected when taking into account neural conduction time. The cABR onset is analogous to wave V of the brainstem's response to a click stimulus, but the cABR has potentially greater diagnostic sensitivity for certain clinical populations. For example, in a comparison between children with learning impairments versus children who are typically developing, significant differences were found for the cABR but not for responses to click stimuli [15]. The FFR comprises two regions: the transition region corresponding to the consonant-vowel (CV) formant transition and the steady-state region corresponding to the relatively unchanging vowel. The CV transition is perceptually vulnerable [16], particularly in noise, and the transition may be more degraded in noise than the steady state, especially in individuals with poorer speech-in-noise (SIN) perception [17].

**Figure 1:**
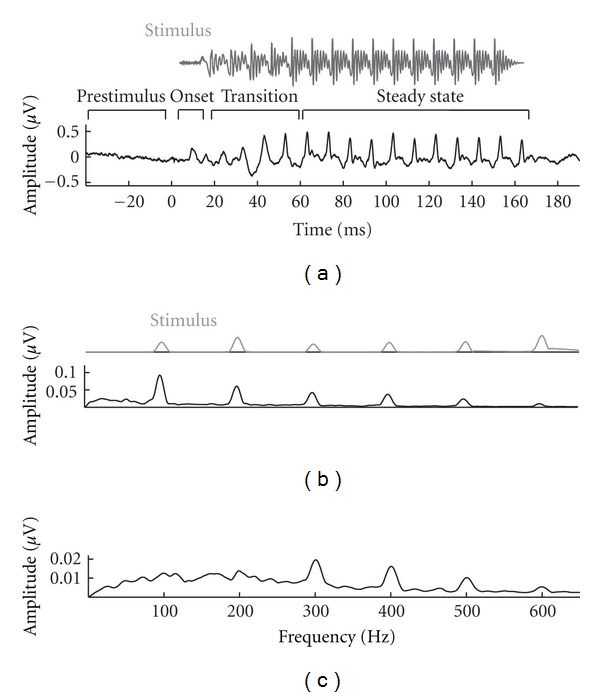
The stimulus /da/ (gray) is displayed with its response (black) in time and frequency domains. (a) Time domain. The response represents an average of 17 older adults (ages 60 to 67) all of whom have audiometrically normal hearing. The periodicity of the stimulus is reflected in the response with peaks repeating every ~10 ms (the *F*_0_ of the vowel /a/). (b) and (c) Frequency domain. Fast Fourier transforms were calculated over the steady-state region of the response, showing frequency energy at the *F*_0_ (100 Hz) and its integer harmonics for responses obtained by adding (b) and subtracting (c) responses to alternating polarities.

The cABR is recorded to alternating polarities, and the average response to these polarities is added to minimize the cochlear microphonic and stimulus artifact [18, 19]. Phase locking to the stimulus envelope, which is noninverting, enhances representation of the envelope and biases the response towards the low frequency components of the response. On the other hand, phase locking to the spectral energy in the stimulus follows the inverting phase of the stimulus; therefore, adding responses to alternating polarities cancels out much of the spectral energy [13, 20]. Subtracting responses to alternating polarities, however, enhances the representation of spectral energy while minimizing the response to the envelope. One might choose to use added or subtracted polarities, or both, depending on the hypothetical question. For example, differences between good and poor readers are most prominent in the spectral region corresponding to the first formant of speech and are therefore more evident in subtracted polarities [21]. In contrast, the neural signature of good speech-in-noise perception is in the low frequency component of the response, which is most evident with added polarities [22]. The average response waveform of 17 normal hearing older adults (ages 60 to 67) and its evoking stimulus and stimulus and response spectra (to added and subtracted polarities) are displayed in Figure 1.

The cABR is acoustically similar to the stimulus. That is, after the cABR waveform has been converted to a  .wav file, untrained listeners are able to recognize monosyllabic words from brainstem responses evoked by those words [23]. The fidelity of the response to the stimulus permits evaluation of the strength of subcortical encoding of multiple acoustic aspects of complex sounds, including timing (onsets, offsets), pitch (the fundamental frequency, *F*_0_), and timbre (the integer harmonics of the *F*_0_) [13]. Analyses of the cABR include measurement of latency and amplitude in the time domain and magnitude of the *F*_0_ and individual harmonics in the frequency domain. Because of the cABR's remarkable stimulus fidelity, cross-correlation between the stimulus and the response also provides a meaningful measure [24]. In addition, responses between two conditions can be cross-correlated to determine the effects of a specific condition such as noise on a response [25].

**Figure 2:**
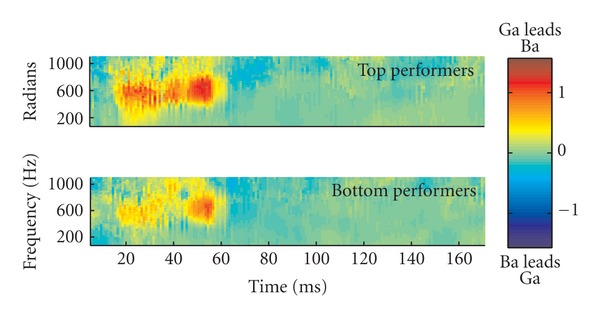
A phaseogram displaying differences in phase (radians, colorbar) in responses to /ba/ and /ga/ syllables, which have been synthesized so that they differ only in the second formant of the consonant-to-vowel transition. The top and bottom groups are children (ages 8 to 12) who differ on a speech-in-noise perception measure, the Hearing in Noise Test (HINT). The red color indicates greater phase difference, with /ga/ preceding /ba/, as expected given cochlear tonotopicity. Note that phase differences are only present in the transition, not in the steady state, during which the syllables are identical. Modified from [27].

Latency analysis has traditionally relied on picking individual peaks, a subjective task that is prone to error. Phase analysis provides an objective method for assessing temporal precision. Because the brainstem represents stimulus frequency differences occurring above 2000 Hz (the upper limits of brainstem phase locking) through timing [26] and phase representation [27, 28], the phase difference between two waveforms (in radians) can be converted to timing differences and represented in a “phaseogram.” This analysis provides an objective measure of the response timing on a frequency-specific basis. For example, the brainstem's ability to encode phase differences in the formant trajectories between syllables such as /ba/ and /ga/ can be assessed and compared to a normal standard or between groups in a way that would not be feasible if the analysis was limited to peak picking (Figure 2). Although the response peaks corresponding to the *F*_0_ are discernible, the peaks in the higher frequency formant transition region such as in Figure 2 would be difficult to identify, even for the trained eye.

In natural speech, frequency components change rapidly, and a pitch tracking analysis can be used to evaluate the ability of the brainstem to encode the changing fundamental frequency over time. From this analysis, a measure of pitch strength can be computed using short-term autocorrelation, a method which determines signal periodicity as the signal is compared to a time-shifted copy of itself. Pitch-tracking error is determined by comparing the stimulus *F*_0_ with the response *F*_0_ for successive periods of the response [29, 30]. These and other measures produced by the pitch-tracking analysis reveal that the FFR is malleable and experience dependent, with better pitch tracking in individuals who have heard changing vowel contours or frequency sweeps in meaningful contexts, such as in tonal languages or music [24, 31].

Other automated analyses which could potentially be incorporated into a clinical protocol include the assessment of response consistency and phase locking. Response consistency provides a way of evaluating trial-to-trial within-subject variability, perhaps representing the degree of temporal jitter or asynchronous neural firing that might be seen in an impaired or aging auditory system [6]. Auditory neuropathy spectrum disorder would be an extreme example of dyssynchronous neural firing, affecting even the response to the click [32–34]. A mild form of dyssynchrony, however, may not be evident in the results of the typical audiologic or ABR protocol but might be observed in a cABR with poor response consistency. The phase-locking factor is another measure of response consistency, providing a measure of trial-to-trial phase coherence [35, 36]. Phase locking refers to the repetitive neural response to periodic sounds. While response consistency is determined largely by the stimulus envelope, the phase-locking factor is a measure of consistency of the stimulus-evoked oscillatory activity [37].

## 3. The cABR and Assessment of Hearing Loss and the Ability to Hear in Noise

The cABR may potentially play an important role in assessment of hearing loss and hearing in noise. It has good test-retest reliability [39, 40], a necessity for clinical comparisons and for documentation of treatment outcomes. Just as latency differences of 0.2 ms for brainstem responses to click stimuli can be considered clinically significant when screening for vestibular schwannomas [9], similar differences on the order of fractions of milliseconds in the cABR have been found to reliably separate clinical populations [41, 42]. Banai et al. [41] found that the onset and other peaks in the cABR are delayed 0.2 to 0.3 ms in children who are good readers compared to poor readers. In older adults, the offset latency is a strong predictor of self-assessed SIN perception in older adults, with latencies ranging from 47 to 51 ms in responses to a 40 ms /da/ (formant transition only) [43]. Temporal processing deficits are also seen in children with specific language impairment, who have decreased ability to track frequency changes in tonal sweeps, especially at faster rates [44]. 

Because of the influence of central and cognitive factors on speech-in-noise perception, the pure-tone audiogram, a largely peripheral measure, does not adequately predict the ability to hear in background noise, especially in older adults [45–47]. Due to the convergence of afferent and efferent transmission in the inferior colliculus (IC) [48, 49], we propose that the cABR is an effective method for assessing the effects of sensory processing and higher auditory function on the IC. While the cABR does not directly assess cognitive function, it is influenced by higher-level processing (e.g., selective attention, auditory training). The cABR is elicited passively without the patient's input or cooperation beyond maintaining a relaxed state, yet it provides in essence a snapshot in time of auditory processing that reflects both cognitive (auditory memory and attention) and sensory influences.

**Figure 3:**
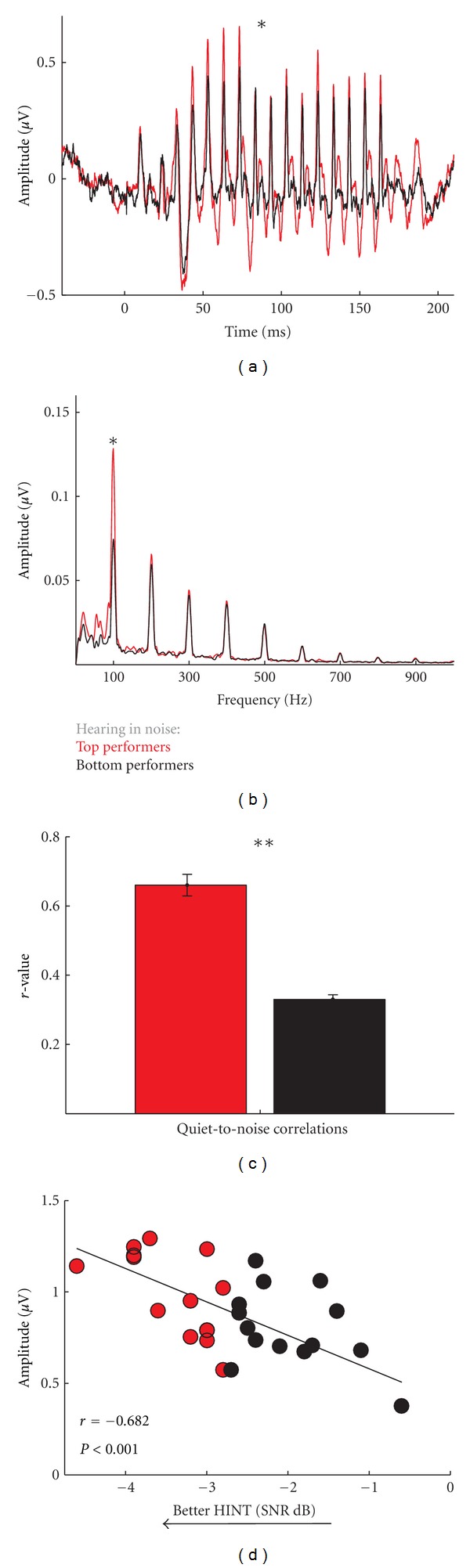
Responses to the syllable /da/ are more robust in older adults with good speech-in-noise perception compared to those with poor speech-in-noise perception, demonstrated by greater RMS amplitude (a) and amplitude of the *F*_0_ in the good speech-in-noise group (b). The responses in the poor speech-in-noise group were more susceptible to the degrading effects of noise, as shown by greater differences in responses to the /da/ in quiet and noise (cross-correlations) (c). Relationship between speech-in-noise perception and the quiet-noise correlation (d). *P < 0.05, **P < 0.01. Modified from [38].

In a study of hearing-, age-, and sex-matched older adults (ages 60–73) with clinically normal hearing, the older adults with good speech-in-noise perception had more robust subcortical stimulus representation, with higher root-mean-square (RMS) and *F*_0_ amplitudes compared to older adults with poor speech-in-noise perception (Figure 3) [38]. Perception of the *F*_0_ is important for object identification and stream segregation, allowing us to attend to a single voice from a background of voices [50]; therefore, greater representation of the *F*_0_ in subcortical responses may enhance one's ability to hear in noise. When we added noise (six-talker babble) to the presentation of the syllable, we found that the responses of individuals in the top speech-in-noise group were less degraded than in the bottom speech-in-noise group (Figure 3). These results are consistent with research from more than two decades documenting suprathreshold deficits that cannot be identified by threshold testing [46, 47, 51–58]. Even in normal-hearing young adults, better speech-in-noise perception is related to more robust encoding of the *F*_0_ in the cABR [53]. Furthermore, in a study with young adult participants, Ruggles et al. [51] found that spatial selective auditory attention performance correlates with the phase locking of the FFR to the speech syllable /da/. Furthermore, they found that selective attention correlates with the ability to detect frequency modulation but is not related to age, reading span, or hearing threshold.

The cABR provides evidence of age-related declines in temporal and spectral precision, providing a neural basis for speech-in-noise perception difficulties. In older adults, delayed neural timing is found in the region corresponding to the CV formant transition [59, 60], but timing in the steady-state region remains unchanged. Importantly, age-related differences are seen in middle-aged adults as young as 45, indicating that declines in temporal resolution are not limited to the elderly population. Robustness of frequency representation also decreases with age, with the amplitude of the fundamental frequency declining in middle- and in older-aged adults. These results provide neural evidence for the finding of adults having trouble hearing in noise as soon as the middle-aged years [61].

**Figure 4:**
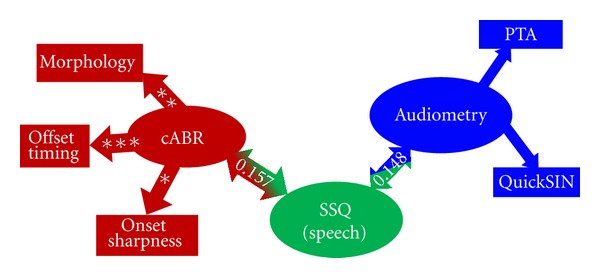
Self-perception of speech, assessed by the Speech Spatial Qualities Hearing scale (SSQ), is predicted by audiologic and cABR measures. The audiometric variables predict 15% of the variance in SSQ; the cABR variables predict an additional 16%. In the multiple linear regression model, only the contributions of the cABR onset time and morphology variables are significant. *P < 0.05, ***P < 0.01.

What is the role of the cABR in clinical practice? The cABR can be collected in as little as 20 minutes, including electrode application. Nevertheless, even an additional twenty minutes would be hard to add to a busy practice. To be efficacious, the additional required time must yield information not currently provided by the existing protocol. One of the purposes of an audiological evaluation is to determine the factors that contribute to the patient's self-perception of hearing ability. To evaluate the effectiveness of possible factors, we used multiple linear regression modeling to predict scores on the speech subtest of the Speech, Spatial, and Qualities Hearing Scale [62]. Pure-tone thresholds, speech-in-noise perception, age, and timing measures of the cABR served as meaningful predictors. Behavioral assessments predicted 15% of the variance in the SSQ score, but adding brainstem variables (specifically the onset slope, offset latency, and overall morphology) predicted an additional 16% of the variance in the SSQ (Figure 4). Therefore, the cABR can provide the clinician with unique information about biological processing of speech [43].

## 4. The cABR is Experience Dependent

As the site of intersecting afferent and efferent pathways, the inferior colliculus plays a key role in auditory learning. Indeed, animals models have demonstrated that the corticocollicular pathway is essential for auditory learning [63, 64]. Therefore, it is reasonable to expect that the cABR reflects evidence of auditory training; in fact, the cABR shows influences of both life-long and short-term training. For example, native speakers of tonal languages have better brainstem pitch tracking to changing vowel contours than speakers of nontonal languages [24]. Bilingualism provides another example of the auditory advantages conferred by language expertise. Bilingualism is associated with enhanced cognitive skills, such as language processing and executive function, and it also promotes experience-dependent plasticity in subcortical processing [65]. Bilingual adolescents, who reported high English and Spanish proficiency, had more robust subcortical encoding of the *F*_0_ to a target sound presented in a noisy background than their age-, sex-, and IQ-matched monolingual peers. Within the bilingual group, a measure of sustained attention was related to the strength of the *F*_0_; this relation between attention and the *F*_0_ was not seen in the monolingual group. Krizman et al. [65] proposed that diverse language experience heightens directed attention toward linguistic inputs; in turn, this attention becomes increasingly focused on features important for speaker identification and stream segregation in noise, such as the *F*_0_.

Musicianship, another form of auditory expertise, also extends to benefits of speech processing; musicians who are nontonal language speakers have enhanced pitch tracking to linguistically relevant vowel contours, similar to that of tonal language speakers [31]. Ample evidence now exits for the effects of musical training on the cABR [28, 60, 67–73]. The OPERA (Overlap, Precision, Emotion, Repetition, and Attention) hypothesis has been proposed as the mechanism by which music engenders auditory system plasticity [74]. For example, there is overlap in the auditory pathways for speech and music, explaining in part the musician's superior abilities for neural speech-in-noise processing. The focused attention required for musical practice and performance results in strengthened sound-to-meaning connections, enhancing top-down cognitive (e.g., auditory attention and memory) influences on subcortical processing [75].

Musicians' responses to the cABR are more resistant to the degradative effects of noise compared to nonmusicians [68, 73]. Background noise delays and reduces the amplitude of the cABR [76]; however, musicianship mitigates the effects of six-talker babble noise on cABR responses in young adults, with earlier peak timing of the onset and the transition in musicians compared to nonmusicians. Bidelman and Krishnan [73] evaluated the effects of reverberation on the FFR and found that reverberation had no effect on the neural encoding of pitch but significantly degraded the representation of the harmonics. In addition, they found that young musicians had more robust responses in quiet and in most reverberation conditions. Benefits of musicianship have also been seen in older adults; when comparing effects of aging in musicians and nonmusicians, the musicians did not have the expected age-related neural timing delays in the CV transition indicating that musical experience offsets the effects of aging [60]. These neural benefits in older musicians are accompanied by better SIN perception, temporal resolution, and auditory memory [77].

But, what about the rest of us who are not able to devote ourselves full time to music practice—can musical training improve our auditory processing as well? Years of musical training in childhood are associated with more robust responses in adults [67], in that young adults with zero years of musical training had responses closer to the noise floor compared to groups of adults with one to five or six to eleven years of training who had progressively larger signal-to-noise ratios. In a structural equation model of the factors predicting speech-in-noise perception in older adults, two subsets were compared—a group who had no history of musical training and another group who had at least one year of musical training (range 1 year to 45 years). Cognitive factors (memory and attention) played a bigger role in speech-in-noise perception in the group with musical training, but life experience factors (physical activity and socioeconomic status) played a bigger role in the group with no experience. Subcortical processing (pitch encoding, harmonic encoding, and cross-correlations between responses in quiet and noise) accounted for a substantial amount of the variance in both groups [78].

Short-term training can also engender subcortical plasticity. Carcagno and Plack [79] found changes in the FFR after ten sessions of pitch discrimination training that took place over the course of approximately four weeks. Four groups participated in the experiment: three experimental groups (static tone, rising tone, and falling tone) and one control group. Perceptual learning occurred for the three experimental groups, with effects somewhat specific to the stimulus used in training. These behavioral improvements were accompanied by changes in the FFR, with stronger phase locking to the *F*_0_ of the stimulus, and changes in phase locking were related to changes in behavioral thresholds.

Just as long-term exposure to tonal language leads to better pitch tracking to changing vowel contours, just eight days of vocabulary training on words with linguistically relevant contours resulted in stronger encoding of the *F*_0_ and decreases in the number of pitch-tracking errors [29]. The participants in this study were young adults with no prior exposure to a tonal language. Although the English language uses rising and falling pitch to signal intonation, the use of dipping tone would be unfamiliar to a native English speaker, and, interestingly, the cABR to the dipping tone showed the greatest reduction in pitch-tracking errors.

Training that targets speech-in-noise perception has also shown benefits at the level of the brainstem [80]. Young adults were trained to discriminate between CV syllables embedded in a continuous broad-band noise at a +10 dB signal-to-noise ratio. Activation of the medial olivocochlear bundle (MOCB) was monitored during the five days of training through the use of contralateral suppression of evoked otoacoustic emissions. Training improved performance on the CV discrimination task, with the greatest improvement occurring over the first three training days. A significant increase in MOCB activation was found, but only in the participants who showed robust improvement (learners). The learners showed much weaker suppression than the nonlearners on the first day; in fact, the level of MOCB activation was predictive of learning. This last finding would be particularly important for clinical purposes—a measure predicting benefit would be useful for determining treatment candidacy.

There is renewed clinical interest in auditory training for the management of adults with hearing loss. Historically, attempts at auditory training had somewhat limited success, partly due to constraints on the clinician's ability to produce perceptually salient training stimuli. With the advent of computer technology and consumer-friendly software, auditory training has been revisited. Computer technology permits adaptive expansion and contraction of difficult-to-perceive contrasts and/or unfavorable signal-to-noise ratios. The Listening and Communication Enhancement program (LACE, Neurotone, Inc., Redwood City, CA) is an example of an adaptive auditory training program that employs top-down and bottom-up strategies to improve hearing in noise. Older adults with hearing loss who underwent LACE training scored better on the Quick Speech in Noise test (QuickSIN) [81] and the hearing-in-noise test (HINT) [82]; they also reported better hearing on self-assessment measures—the Hearing Handicap Inventory for the Elderly/Adults [83] and the Client Oriented Scale of Improvement [84, 85]. The control group did not show improvement on these measures.

**Figure 5:**
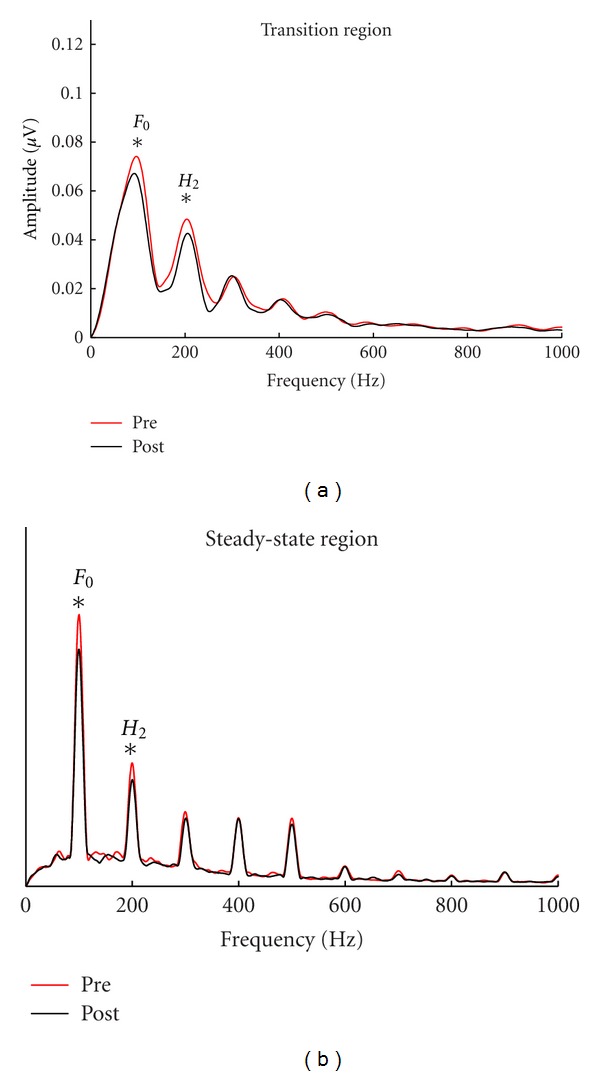
Young adults with normal hearing have greater representation of the *F*_0_ in subcortical responses to /da/ presented in noise after undergoing LACE auditory training. The *F*_0_ and the second harmonic have greater amplitudes in the postcondition when calculated over the transition (20–60 ms) (b) and the steady state (60–170 ms) (a). Modified from [66].

The benefits on the HINT and QuickSIN were replicated in young adults by Song et al. [66]. After completing 20 hours of LACE training over a period of four weeks, the participants improved not only on speech-in-noise performance but also had more robust speech-in-noise representation in the cABR (Figure 5). They had training-related increases in the subcortical representation of the *F*_0_ in response to speech sounds presented in noise but not in quiet. Importantly, the amplitude of the *F*_0_ at pretest predicted training-induced change in speech-in-noise perception. The advantages of computer-based auditory training for improved speech-in-noise perception and neural processing have also been observed in older adults [86]. Based on this evidence, the cABR may be efficacious for documenting treatment outcomes, an important component of evidence-based service.

## 5. The cABR and Hearing Aid Fitting

Any clinician who has experience with fitting hearing aids has encountered the patient who continues to report hearing difficulties, no matter which particular hearing aid or algorithm is tried. Although we have not yet obtained empirical evidence on the role of the cABR in the hearing aid fitting, we suggest that implementation of the cABR may enhance hearing aid fittings, especially in these difficult-to-fit cases. The clinician might be guided in the selection of hearing aid algorithms through knowledge of how well the brainstem encodes temporal and spectral information. For example, an individual who has impaired subcortical timing may benefit from slowly changing compression parameters in response to environmental changes.

**Figure 6:**
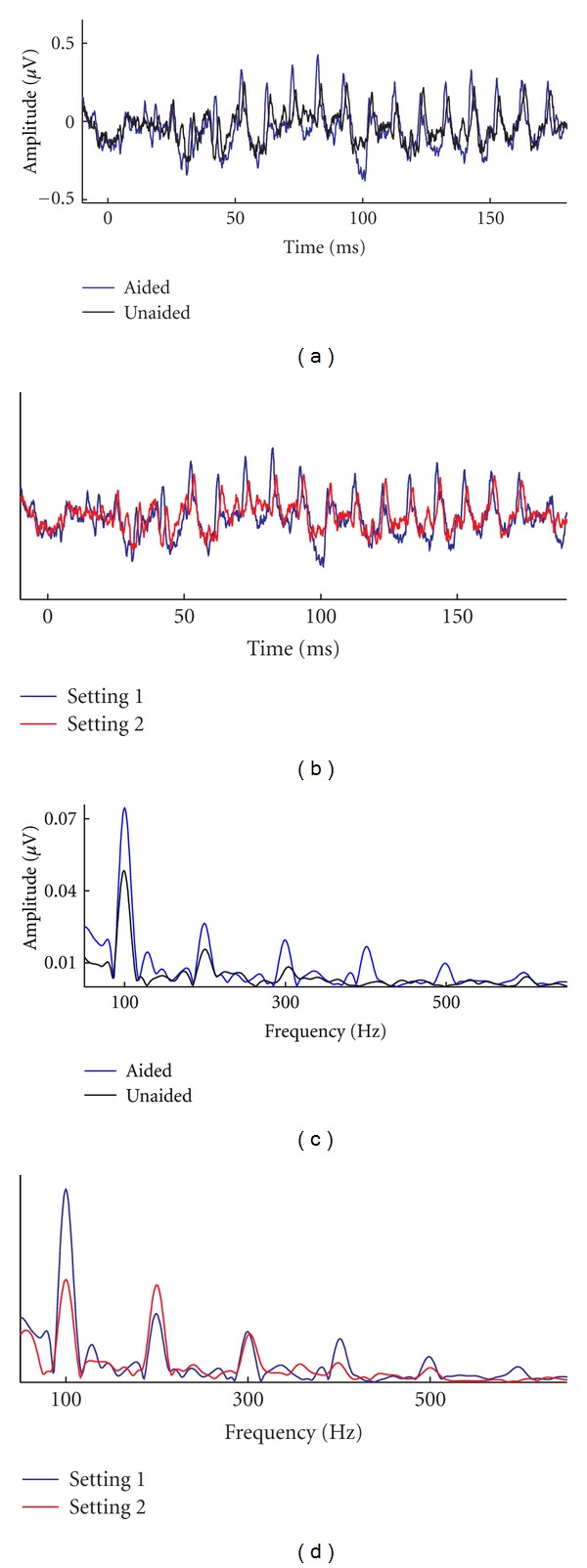
Responses were obtained to the stimulus /da/ presented at 80 dB SPL in sound field in aided (blue) versus unaided (black) conditions ((a) and (c)) and different settings in the same hearing aid ((b) and (d)). Responses show greater RMS and *F*_0_ amplitudes in aided versus unaided conditions and for setting 1 versus setting 2.

We envision incorporating the cABR into verification of hearing aid performance. Cortical-evoked potentials have been used for verifying auditory system development after hearing aid or cochlear implant fitting in children [87–89]. In adults, however, no difference is noted in the cortical response between unaided and aided conditions, indicating that the cortical response may reflect signal-to-noise ratio rather than increased gain from amplification [90]. Therefore, cortical potentials may have limited utility for making direct comparisons between unaided and aided conditions in adults. We recently recorded the cABR in sound field and compared aided and unaided conditions and different algorithms in the aided condition. There is a marked difference in the amplitude of the waveform in response to an aided compared to an unaided condition. By performing stimulus-to-response correlations, it is possible to demonstrate that certain hearing aid algorithms resulted in a better representation of the stimulus than others (Figure 6). These preliminary data demonstrate the feasibility and possibility of using this approach. Importantly, these data also demonstrate meaningful differences easily observed in an individual.

## 6. Conclusions

With improvements in digital hearing aid technology, we are able to have greater expectations for hearing aid performance than ever before, even in noisy situations [91]. These improvements, however, do not address the problems we continue to encounter in challenging hearing aid fittings that leave us at a loss for solutions. The cABR provides an opportunity to evaluate and manage an often neglected part of hearing—the central auditory system—as well as the biological processing of key elements of sound. We envision future uses of the cABR to include assessment of central auditory function, prediction of treatment or hearing aid benefit, monitoring treatment or hearing aid outcomes, and assisting in hearing aid fitting. Because the cABR reflects both sensory and cognitive processes, we can begin to move beyond treating the ear to treating the person with a hearing loss.
